# Smoking among school-going adolescents in selected secondary schools in Peninsular Malaysia- findings from the Malaysian Adolescent Health Risk Behaviour (MyaHRB) study

**DOI:** 10.1186/s12971-016-0108-5

**Published:** 2017-01-31

**Authors:** Kuang Hock Lim, Hui Li Lim, Chien Huey Teh, Chee Cheong Kee, Yi Yi Khoo, Shubash Shander Ganapathy, Miaw Yn Jane Ling, Sumarni Mohd Ghazali, Eng Ong Tee

**Affiliations:** 1 Institute for Medical Research, Jalan Pahang, 50588 Kuala Lumpur, Malaysia; 2 Melaka Manipal Medical College, Jalan Batu Hampar, Kuala Lumpur, 75150 Melaka Malaysia; 3 Institute for Public Health, Jalan Bangsar, 50590 Kuala Lumpur, Malaysia; 4 Allied Health College, Jalan Hospital, 47000 Sg. Buloh, Malaysia

**Keywords:** Adolescent smoking, Intrapersonal, Interpersonal, School-going adolescents, Peninsular Malaysia

## Abstract

**Background:**

A multitude of studies have revealed that smoking is a learned behaviour during adolescence and efforts to reduce the incidence of smoking has been identified as long-term measures to curb the smoking menace. The objective of this study was to determine the prevalence as well as the intra and inter-personal factors associated with smoking among upper secondary school students in selected schools in Peninsular Malaysia.

**Methods:**

A study was carried out in 2013, which involved a total of 40 secondary schools. They were randomly selected using a two-stage clustering sampling method. Subsequently, all upper secondary school students (aged 16 to 17 years) from each selected school were recruited into the study. Data was collected using a validated standardised questionnaire.

**Results:**

This study revealed that the prevalence of smoking was 14.6% (95% CI:13.3–15.9), and it was significantly higher among males compared to females (27.9% vs 2.4%, *p* < 0.001). Majority of smokers initiated smoking during their early adolescent years (60%) and almost half of the respondents bought cigarettes themselves from the store. Multivariable analysis revealed that the following factors increased the likelihood of being a current smoker: being male (aOR 21. 51, 95% CI:13.1–35), perceived poor academic achievement (aOR 3.42, 95% CI:1.50–7.37) had one or both parents who smoked (aOR 1.80, 95% CI:1.32–2.45; aOR 6.50, 95 CI%:1.65–25.65), and always feeling lonely (aOR 2.23, 95% CI:1.21–4.43). In contrast, respondents with a higher religiosity score and protection score were less likely to smoke (aOR 0.51, 95% CI:0.15–0.92; aOR 0.71, 95% CI 0.55–0.92).

**Conclusion:**

This study demonstrated that the prevalence of smoking among Malaysian adolescents of school-going age was high, despite implementation of several anti-smoking measures in Malaysia. More robust measures integrating the factors identified in this study are strongly recommended to curb the smoking epidemic among adolescents in Malaysia.

## Background

Mortality and morbidity due to smoking-related diseases are important public health issue globally [1]. The World Health Organization (WHO) reported that mortality due to smoking-related diseases was higher than the combination of all infectious diseases worldwide. By 2030, if there is no change in the trend, the mortality rate due to smoking will have increased by 2.5 fold, whereby 70% of this rate will be from developing and under-developed countries. In Malaysia, approximately 10,000 deaths attributed to smoking were reported annually [2], with 5.6 million years of life lost (YLLs) [3] and almost three billion Malaysian Ringgit (RM) had been spent to treat three smoking-related diseases, namely chronic obstructive pulmonary disease (COPD), ischemic heart disease (IHD) and lung cancer [4]. In addition, studies had also demonstrated that smokers were more likely to indulge in high-risk health behaviours such as use of illicit drugs [5], pre-marital sex [6] and alcohol usage [7].

A plethora of studies have shown that majority of adult smokers initiated smoking during their adolescent years [8, 9], and they were more inclined to continue this habit into their adulthood [9] and more likely to be afflicted by smoking-related diseases and less likely to cease smoking [10]. In order to prevent smoking initiation at young age, determination of correlates of adolescent smoking is of paramount importance for anti-smoking policy planning and implementation.

Studies have shown that smoking is a complex behaviour. Some of the identified contributory factors to adolescent smoking include intra- and inter-personal factors and environmental factors [11-18]. Although research on smoking among adolescents in Malaysia had been carried out over the past few decades, these studies, including the nationwide studies were mainly concentrated on prevalence and psychosocial factors [11, 12, 15–17]. Intrapersonal factors such as unsatisfactory academic achievement and religiosity were not given due attention. Therefore, this manuscript aimed to address the knowledge gaps by determining the prevalence of smoking, characteristics of smokers, as well as inter and intra-personal factor(s) associated with smoking among upper secondary school adolescents (aged 16 to 17 years old) in selected schools in Peninsular Malaysia. These findings could provide evidence-based findings to enable the formulation and implementation of suitable policies to curb the increasing burden of smoking-related morbidity and mortality in Malaysia, especially amongst the smoking adolescents aged 16 to 17 years.

## Methods

The MyAHRB study was a cross-sectional study conducted in 11 states involving 20 districts in Peninsular Malaysia from May to September 2013. Two-stage proportionate-to-size sampling technique was utilised to obtain the sample of schools; the first stage involved the selection of districts with Clinical Training Centres (CTCs) for public health paramedics, followed by stratification of schools by locality (urban/rural). Two secondary schools were then randomly selected from each district via simple random sampling method. As a result, a total of 40 schools were selected (20 schools in urban and 20 in rural area). All students aged 16–17 years from the selected schools were recruited as participants. Students of non-Malaysian citizenship were excluded from the study. The sample size was determined using an estimated prevalence of suicidal ideation from pre-test (3%), a design effect of 3 to cater for clustering effect from each school, a precision of 1.5% and a non-response rate of 20%. Based on this parameters, a total of 3578 respondents were needed for the study.

### Study instrument

A validated self-administered questionnaires was used in the MyAHRB study. The structured questionnaire consisted of four sections: sociodemography (age, gender and ethnicity), self-perceived academic achievement, parent/s’ educational level, parent/s’ occupation, household size, parents’ marital status (married/divorced), health-risk behaviour [alcohol consumption, sexual behaviour], Rosenberg Self-Esteem Scale and religiosity.

Health-risk behaviours were assessed using a questionnaire adapted from the Global School-based Student’s Health Survey (GSHS) [19] and the Youth Risk Behaviour Surveillance (YBRS) [20]. The health-risk behaviour items were translated by a panel of experts consisting of language and content experts, using backward and forward translation processes. The Rosenberg Self-Esteem Scale was adopted from Jamil [21] whereas the religiosity items were adopted from a health behaviour study questionnaire developed by University Putra Malaysia. The questionnaire was pre-tested and further modified based on feedback from selected students in certain schools in Kuala Lumpur to suit the local socio-cultural context.

The permission to conduct the MyaHRB study was granted by the Ministry of Education and State Education Department. Ethical approval was obtained from the Malaysia Research Ethics Committee, Ministry of Health Malaysia.

### Measures

Consent was obtained from parents/guardians of the selected students. The consent form provided information about the participation of their children in the study, as well as the study objectives. Participations of students were of voluntary basis and parents/guardians of the selected respondents were asked to return the consent form if they did not agree for their son/daughter to participate in the study. Only respondents who did not return the consent form were allowed to participate in the study. Data collection was carried out in the designated area allocated by the school administration. Staff and teachers were not allowed to be around during the questionnaire-answering session to avoid “Hawthorne effect”. Briefing was given by the team members before the session began. This included the objectives of the study, the anonymity of the answers given, awareness that their participation was on a voluntarily basis, as well as an explanation of the items in the questionnaire. Respondents were also requested not to write their names or provide any information that would reveal their identities, with the exception of their signatures which indicated their willingness to participate in the study. Respondents who did not understand certain items in the questionnaire were assisted by the research team members. All completed questionnaires were sealed in envelopes.

The dependent variable in this questionnaire was “current smoker”, which was evaluated using the item “In the last 30 days, how often did you smoke?”. Respondents who answered “every day”, “almost every day”, “2–3 times a week”, “once a week” and “once a month” were classified as “current smoker” whilst those who answered “I did not smoke” during the last 30 days were categorized as “non-smoker”. Those who answered “smoked at least once a month” were required to answer their age of smoking initiation, quantity of cigarettes smoked per day and source of cigarettes. Those who smoked less than 11 sticks per day was classified as light smoker, 11–20 sticks as moderate smoker and more than 20 sticks per day as heavy smoker. The independent variables included “parents/guardian who smoked”, “perception of academic achievement “, family status (whether their parents were married/divorced), and education attainment of their parents. A validated Malay version of the Rosenberg Self-Esteem Scale was used to evaluate the level of self-esteem, whereby those who scored less than 15 were categorized as having “low self-esteem” and 15–30 as having “high self-esteem”. The protection factor was evaluated using 6 items (for example “My parents know what I am doing during my free time”, “friends always help me in school”). Religiosity was examined using three items (for example “Do you agree that religion is very important to guide your life?”). Both these variables were measured using the Likert-type scale. A higher score indicated a higher protective factor in school and within their families as well as portrayed the importance of religion in daily life, respectively. The number of close friend(s) was measured using the item “How many close best friends do you have?” with the option “None, One, Two”. Questions on how often they felt lonely was evaluated using the item “In the last one month how often do you feel lonely?” with the choice of “Always or sometimes”.

### Data analysis

The data were cleaned before the analysis, whereby outlier values were detected using frequency analysis and references were made against the original questionnaire if the investigators had any doubt about the answers given. Descriptive statistics was used to illustrate the demographic status of the respondents, age of smoking initiation, number of cigarettes smoked and source of cigarettes. Chi-square analysis was used to determine the association between smoking status and social demographic variables whilst independent *T*-test was used to determine the mean differences of protection and religiosity scores between smokers and non-smokers. All independent variables with *p* < 0.25 in univariate analysis (Chi-square and *T*-test) were included in the Multivariable Logistic Regression model to determine the effect of each independent variable after controlling the influence of other independent variables. A co-linearity test between religiosity and protection score was carried out by variant inflation factor and the value of 1.025 indicated no co-linearity between the two variables. A Hosmer–Lemeshow value of 0.25 indicated the fitness of the model. All possible two-way interactions between the independent variables in the final model were also analysed. No interaction with *p* < 0.05 were detected, indicating no significant two-way interactions. All statistical analyses were run at 95% confidence interval using SPSS software version 16.

## Results

A total of 2991 respondents participated in this study which led to an overall response rate of 83.6%. Out of 2991 participants, 2794 of them responded to the question on smoking module, giving a response rate of 93.4% to this section. The students were composed almost equally by gender [51.8% (1448/2794) were females and 48.2% (1346/2794) were males). The largest ethnic group likening to more than three-quarters of the respondents was Malays, followed by Chinese (14.8%). The prevalence of smoking was 14.6% (408/2793) (95% CI:13.3–15.9) and this was higher among male students as compared to females (27.9% vs 2.4% *p* < 0.001). Smoking prevalence was also significantly higher among the Malays, those who perceived their academic achievement as poor, who always felt lonely, had parent/s or guardian/s who smoked and with two best friends (Table 1).

**Table 1:** Prevalence of smoking among upper secondary school students in Peninsular Malaysia

Variable	Smoking		Chi-square value	*p* value
	Yes	No		
	N(%)	N(%)		
Gender				
Male	373(27.9)	963(72.1)	361.3	<0.001
Female	35(2.4)	1413(97.6)		
Ethnicity				
Malay	372(17.2)	1812(82.8)	59.2	<0.001
Chinese	18(4.6)	375(95.4)		
Indian	10(5.1)	186(94.9)		
Others	3(27.3)	8 (72.7)		
Academic Achievement				
Excellence	194(11.3)	1516(88.7)	51.7	<0.001
Moderate	181(18.9)	779(81.1)		
Unsatisfactory	32(32.4)	70(68.6)		
Marital status of parents				
Married	380(14.5)	2233(85.5)	0.01	0.79
Divorced	23(15.3)	127(84.7)		
Level of self esteem				
High	192(16.3)	988(83.7)	6.37	0.012
Low	189(12.8)	1288(87.2)		
Loneliness				
Always	37(21.3)	137(78.7)	6.52	0.01
Sometimes	370(14.2)	2236(85.8)		
Number of parents Smoked				
None	209(11.1)	1670(88.9)	40.69	<0.001
One	145(18.8)	627(81.2)		
Both	8(42.1)	11(57.9)		
Number of Best friend/s				
None	10(9.3)	97(90.3)	2.56	0.27
One	14(13.2)	91(86.8)		
Two	380(14.8)	218(85.2)		

Table 2 highlighted that almost 70% of current smokers bought cigarettes from the premises (i.e., sundry shops) by themselves and more than one-third of them smoked daily. Approximately 90% of current smokers were light smokers (smoked less than 11 sticks per day) and almost two-thirds of them started smoking during upper primary or lower secondary school (aged 11–14 year old). Table 3 showed that respondents with high protection and religiosity scores were less likely to smoke in the last 30 days (protection score 3.61 vs 3.42, *p* < 0.001; religiosity score 3.67 vs 3.49, *p* < 0.001).

**Table 2:** Smoking initiation age, number, frequency of smoking and source of cigarettes/s among current adolescent smoking

Variable	n	%
Smoking initiation Age (year)		
Less than 7	14	3.5
8–9	23	5.7
10–11	60	14.9
12–13	125	31.1
14–15	148	36.8
16 years	34	8.5
Quantity of cigarettes daily		
Less than 1	32	7.8
1	72	17.6
2–5	179	43.8
6–10	53	13.0
11–20	22	5.4
> 20	30	7.3
Frequency of smoking in the last 30 days		
Daily	143	34.7
20–29	37	9.0
10–19	40	9.7
6–9	41′	10
3–5	45	10.9
1–2	106	25.7
Source of cigarettes		
Bought from shop	253	69.9
Asked others to buy	29	8.0
Bought from others	45	12.4
Family member	12	3.3
Others	23	6.4

**Table 3:** Mean score for protection and Religiosity between smoking and non-smoking adolescents

Variable	Smoking			
	Yes	No	T score	*p* value
	Mean (sd)	Mean (sd)		
Protection scale	3.42 (0.51)	3.61 (0.59)	5.40	0.001
Religiosity	3.49 (0.52)	3.67 (0.49)	5.76	0.001

Multivariable analysis revealed that the odds of smoking increased among males (aOR 21.51, 95% CI:13.1–35.1), those who perceived poor academic achievement (aOR 3.42, 95% CI:1.50–7.37, excellent group as reference group), had both parents who smoked (aOR1.80, 95% CI:1.32–2.45), had one parent who smoked, (aOR 6.50, 95% CI:1.65–25.65, no parent smoked as reference), had two best friends (aOR 4.40, 95% CI:1.38–14.03, having no best friend as reference) and always felt lonely (aOR 2.23, 95% CI:1.21–4.43). On the other hand, respondents with higher religiosity score and protection score were less likely to smoke (aOR 0.51, 95% CI:0.15–0.92; aOR 0.71, 95% CI:0.55–0.92) (Table 4).

**Table 4:** Factors related to smoking among adolescents using Multivariable Logistic Regression analysis

Variable	Wald Value	Adjusted OR	95 CI
Gender			
Male	150.28	21.51	13.1–35.1
Female		1	
Ethnicity			
Malay		1	
Chinese	28.54	0.09	0.04–0.22
Indian	15.16	0.18	0.07–0.40
Others	0.37	2.22	0.17–27.82
Academic Achievement			
Excellence		1	
Moderate	3.42	1.34	0.98–1.82
Not Good	9.89	3.42	1.50–7.37
Protective factor score	6.77	0.71	0.55–0.92
Religiosity score	20.17	0.51	0.15–0.92
Loneliness			
Always	6.57	2.23	1.21–4.13
Sometimes		1	
Number of parents who smoke			
None		1	
One	13.41	1.80	1.32–2.45
Both	7.14	6.50	1.65–25.65
Number of Best friend/s			
None		1	
One	2.54	3.45	0.75–15.81
Two	6.26	4.40	1.38–14.03
Rosenberg self-esteem scale			
Low	2.56	1.26	0.95–1.66
High	1		

## Discussion

The prevalence of current smokers was 14.6%, which was consistent to the 14.3% reported by Lim KH et al. among adolescents in Petaling District [12]. However, this was almost 3% higher than those reported in a nationwide study conducted in 2012 among secondary school students [11] and a smaller-scaled study among adolescents in Kinta, Perak [22]. In addition, this prevalence was also higher than those reported in Thailand (8.8%) and Philippines (11.0%) [23]. The higher proportion of smoking among school going adolescents in Malaysia might due to the measure/s implemented to address the problem of smoking among adolescent in Malaysia are not as comprehensive and throughout as compared to those countries, On the other hand, the smoking ratio of 10:1 among male and female Malaysian adolescents was comparable with those reported in other local studies as well as several Asian countries [11, 22, 24]. However, it was not in line with those demonstrated in western countries, whereby an almost equal proportion of male and female adolescent smokers was observed [25]. These contradictory findings can be explained by the fact that smoking among females was less likely to be accepted as compared to their male counterparts, especially in the Asian society such as Malaysians. In addition, male adult smokers may also pay a significant role in influencing adolescent males to initiate and adopt smoking behaviour since the Social Learning Theory proposed that learning via observation is more effective among the same gender [26]. Also, unconscious biases and cultural scripts that daughters are in need of more ‘protection’ which led to more parental attention could also be the plausible reason of lower smoking prevalence among Malaysian female adolescents. In contrast, researchers stipulated that the rising prevalence of female smoking in the western regions could be partly due to the change of social norm (permissible society norm) towards smoking among females [27].

The sale of cigarettes to persons aged below 18 year-old is prohibited under the smoking control regulation in Malaysia since 2006. However, almost two-thirds of adolescent smokers in the present study, 65.4% Form Four students (aged 16 years old) in Petaling District, Malaysia [11] and 68.3% Malaysian adolescents [28] reported that they were able to purchase cigarettes from commercial sources. This implies that the law was not being taken seriously and/or the existing enforcement is inadequate [29–31]. This calls for stricter enforcement, especially near education facilities, as studies had showed that effective law enforcement could reduce commercial source of cigarettes [32–34] and ultimately reduce adolescent smoking [35].

This study also found that most adolescents initiated smoking during upper primary or lower secondary schooling period. This finding resonates with those reported by by Lim et al. and studies conducted in developed countries [12, 15, 36, 37]. This might be because adolescents at this age feel that they are constantly at the center of attention and the people surrounding them are inspecting either their appearance or actions. This belief might drive them into conducting risk-taking actions, as such initiating smoking [38]. Nevertheless, future studies are recommended to explore the association or causal effect of adolescents emotion/feeling and smoking behaviour.

The present study demonstrated a dose-response relationship between adolescent smoking and the smoking status of one or two parent(s). The likelihood of smoking increased when both parents smoked. These findings were consistent with those reported elsewhere [14, 39–43]. According to Bandura’s concept of “delayed modelling” [44], during childhood, an individual learns or remembers how to perform behaviour from seeing it modelled by their parents. Therefore, parents who smoked in front of their children would act as a role model for their children and also indirectly provide an impression that smoking is a normative behaviour among adults [45]. The mentally immature adolescents would adopt the smoking behaviour of their parents to satisfy their desire to be like an adult. Furthermore, parents who smoked were usually more liberal when dealing with smoking issues [46] and therefore less likely to convey the hazard of smoking to their children [47], which would ultimately lead to the thinking that smoking is acceptable and permissible by their smoking parents. In addition, smoking parents may also think that they do not have legitimate authority to advice or convince their children not to smoke because they themselves are smokers [48].

Having more best friends had been demonstrated by many studies to be a protection factor against smoking since having more friends would enable the sharing of problems, reducing stress and therefore reduce the likelihood in involving in risky health behaviors such as smoking. However, the present study demonstrated a contradictory finding. This could be partly explained by the Hemophilia Theory posited by Brickers et al. who stated that smoking adolescents tend to be friend with those who also shared similar behavior, i.e., smoking [49]. However, further study is needed to elucidate the actual reason for such association.

The present findings corroborated the well-established evidence that unsatisfactory academic achievement was a significant risk factor associated with adolescent smoking [12, 13, 15, 50]. Good academic achievement might be a manifestation of cognitive gains, which may assist adolescents to understand and underscore the negative impacts of unhealthy behaviors such as smoking, which may then drive them away from the behaviour. In contrast, students who experienced academic failures may less attached to school and may befriend peers who smoked, thus increase the likelihood for them to perform similar behaviour [51, 52].

Adolescents who always felt lonely were more likely to smoke compared to those who did not. This finding was in accordance with a study by Page et al. [53] who reported that lonely boys and girls were more likely to smoke in Chile and Namibia. In addition, Stickly et al. [54] also reported that lonely boys and girls in Russia and the USA had higher odds for engaging in at least one type of risky substance-abuse behaviour. Similar finding was reported by Park [9] among adolescents in Korea. Cigarette use may be a mean to assuage the negative feelings that arose from being lonely [53]. Alternatively, loneliness may be an indication of other psychological problems, such as stress and depression [55, 56], and people usually perceived smoking can alleviate stress, depression and other psychological problems.

The present findings demonstrated that religiosity score was inversely associated with smoking and such findings were consistent with other local studies [13, 57, 58] as well as few other studies in United State of America (USA) [59–61]. This might be explained by the Social Control Theory, which posited that the internalization of religion among adolescents could motivate them to follow the tenets stipulated in their religion. In the present study, a majority of adolescents were Muslims and since Islam does not encourage smoking (Makruh), this may explain the protective effect of high religiosity against smoking. In addition, study had also demonstrated that higher religiosity was positively associated with mental wellbeing and coping mechanisms which are related to cigarette smoking [59]. Besides, adolescents who embraced higher religiosity were more likely to befriend with peers who had similar values [62] hence encouraging and promoting positive behaviour through peer modelling and social support [63].

Respondents who scored higher on the protection score (perception towards attention, family relationships and assistance given by friends in school) were less likely to smoke. This finding was in line with the those reported by Shakib et al. [64] who revealed that a decrease in family concern was the main factor associated with smoking among adolescents in China. In addition, Wen and Shenessa [65] and Wang et al. [66] had also found that adolescents who perceived more attention from parents were less likely to smoke. As adolescents who had a good relationship with their parents tend to be more satisfied with life, more future-oriented and less stressed. Furthermore, assistance by schoolmates might enable adolescents to share their problems and reduce stress, which might help them to avoid risky health behaviour, such as smoking.

### Limitations

This study was not without limitations. First, the cross-sectional nature of the study only allowed determination of the association between dependent and independent variables but not causal relationship. Second, the smoking status of adolescents was self-reported without any biochemical validation such as measurement of cotinine level in urine. Third, the generalization of the findings from this study can only be applied to school-going adolescents aged 16 to 17 years but not to all school-going adolescents in Malaysia. Finally, recall bias especially on several elements such as number of cigarette smoked per day was inevitable.

## Conclusion

In conclusion, this study provides evidence-based findings for the planning and implementation of targeted public health policies to combat the relatively high prevalence of adolescent smoking. Anti-smoking campaigns should concentrate or emphasize more on male adolescents, those of Malay descent, with unsatisfactory academic achievements and had smoking family members and/or peer. Parents/guardians, particularly those who smoked should also be invited to be involved in all anti-smoking activities together with their children in order to serve as a positive role model to discourage non-smoking adolescents from initiating this habit and for smoking adolescents to quit smoking. Last but not least, enforcement activities towards the sale of tobacco products to adolescents and smoking in public areas should be enhanced to prevent smoking initiation and to denormalize smoking as a norm in our society.

## Abbreviations

COPD: Chronic obstructive pulmonary disease
GSHS: Global school-based student’s health survey
IHD: Ischemic heart disease
MyaHRB: Malaysian Adolescent Health Risk Behaviour
YBRS: Youth Risk Behaviour Surveillance
YLL: Years of life lost
